# State-of-the-Art: Inflammatory and Metabolic Markers in Mood Disorders

**DOI:** 10.3390/life10060082

**Published:** 2020-06-06

**Authors:** Federico Mucci, Donatella Marazziti, Alessandra Della Vecchia, Stefano Baroni, Paolo Morana, Barbara Carpita, Paola Mangiapane, Florinda Morana, Benedetto Morana, Liliana Dell’Osso

**Affiliations:** 1 Dipartimento di Medicina Clinica e Sperimentale, Section of Psychiatry, University of Pisa, 56100 Pisa, Italy; dmarazzi@psico.med.unipi.it (D.M.); alessandradellavecchia@gmail.com (A.D.V.); stefanobaroni1967@gmail.com (S.B.); liliana.dellosso@med.unipi.it (L.D.); 2 Dipartimento di Biotecnologie, Chimica e Farmacia, University of Siena, 53100 Siena, Italy; barbara.carpita1986@gmail.com; 3 Casa di Cura Morana, 91025 Marsala, Italy; paolomorana@yahoo.com (P.M.); paola.mangiapane@virgilio.it (P.M.); florindamorana@gmail.com (F.M.); benedettomorana@gmail.com (B.M.)

**Keywords:** mood disorders, neurotransmitters/neuroreceptors, serotonin, immune system, neuro-inflammation, hypothalamus-pituitary-adrenal axis, neurotrophins, uric acid, oxidative stress

## Abstract

Mounting evidence highlights the involvement of inflammatory/immune systems and their relationships with neurotransmitters and different metabolic processes in mood disorders. Nevertheless, there is a general agreement that available findings are still inconclusive. Therefore, further investigations are required, aimed at deepening the role of possible alterations of biomarkers in the pathophysiology of mood disorders that might lead to more focused and tailored treatments. The present study is a comprehensive review on these topics that seem to represent intriguing avenues for the development of real innovative therapeutic strategies of mood disorders.

## 1. Introduction

Mood disorders include different conditions characterized by alterations of mood, psychomotricity, and bio-rhythmicity, provoking great subjective suffering and social and relational impairments [1,2,3,4]. Major depressive disorder (MDD) and bipolar disorders (BDs) are the most severe and common forms of mood disorders [5]. The first is characterized by depressive episodes, while the most typical BD features are alternating mood shifts, of mania or hypomania, with depressive episodes [6,7,8]. The lifetime prevalence of major depressive disorder is around 7% (about 264 million people), and that of bipolar disorders >1% (about 45 million people) [7,8,9]. As noted by the World Health Organization (WHO), it represents the second main cause of disability in 2020, with a critical burden on the worldwide economy [10,11]. Several authors have hypothesized that unipolar and bipolar depression may represent distinct nosological entities, possibly related to different biological substrates, and sought to differentiate them from a phenomenological point of view [12]. Research on this topic is inconclusive, and a probabilistic and dimensional approach (mood spectrum model) prevails today. Therefore, different authors and clinicians tend to identify MDD as a part of a wider continuum, called “bipolar spectrum”, encompassing depressive and manic symptomatology of different severity levels along a longitudinal course. In several patients, the onset of depression may cause them to end up with mania, or, more frequently, depressive symptoms appear mixed with manic symptoms within the same episode [12].

Although mood disorders are one of the most common causes of disability worldwide among psychiatric syndromes [7,10,13], their pathophysiology is still unknown, and their pharmacological management is still at issue, given the evidence that they are chronic conditions, with only 30% of patients achieving complete remission of symptoms. For years, after discovery of the first antidepressants (ADs), the so-called tricyclic ADs, the alteration of monoamine neurotransmission, was considered the most relevant pathophysiological mechanism of MDD [9]. However, data supporting the monoamine hypotheses are inconclusive as they cannot explain the complex clinical picture [9,13,14,15], and Ads, developed on their basis, have shown limited effectiveness. Only two-thirds of MDD patients may benefit from these pharmacological treatments; furthermore, part of the responders relapse after initial improvement, even if the drug is continued [16].

Subsequent studies carried out on ADs and mood stabilizers showed that prolonged exposure might also trigger the activation cascade of different genes involved in expression and synthesis of different trophic factors [17,18,19]. At the same time, it was demonstrated that the plastic properties of neurons are associated with cell morphology changes, with an increase or decrease in synapses and dendritic spine formation [20,21,22], and that the loss of neuronal trophism leads to a reduction in the environmental stimuli adaptation ability of the brain areas regulating emotions. Taken together, these data have contributed to the definition of the "neurotrophic hypothesis", based on the concept of trophism loss in some brain areas after prolonged stressful periods. Experimental studies on rats subjected to chronic stress showed that neurons of the nucleus accumbens, hippocampus, and prefrontal cortex undergo a reduction in the density of the dendritic spines and of the arborization of the synapses, as well as a loss of volume, which is called "shrinking" [23,24]. Shrinking, a phenomenon also observed in depressed patients and in subjects who undergo chronic stress, is significantly improved by ADs [25]. The neurotrophins most involved in mood disorder pathophysiology are the Brain-Derived Neurotrophic Factor (BDNF) and, to a lesser extent, the Glial-Derived Neurotrophic Factor (GNDF). It has been hypothesized that compounds capable of overcoming the blood–brain barrier, and exerting a modulating function on specific genes involved in trophic and/or immune system factors, might represent an opportunity for the development of potential new and more effective psychotropic compounds in the near future. However, the preliminary data on the current experimental compounds are not encouraging due to their severe side effects [26,27].

Besides the monoaminergic and neurotrophic hypotheses, other neurobiological models of mood disorders have been developed throughout the decades, from the hypothalamus-pituitary-adrenal (HPA) axis dysfunctions to the inflammatory/immune system alterations, to the structural or functional abnormalities of emotional circuits (i.e., the so-called “limbic cortical model” and the “cortico-striatal model”) [28,29,30]. Growing evidence highlights the main role of inflammation in mood disorders, which seems to correlate with all of the other proposed mechanisms, such as the neurotrophin, neurotransmitters, and the HPA axis [31,32,33]. 

The purpose of this article is to review the available literature data on the role of inflammation in the pathophysiology of mood disorders, with a particular attention to its interactions with the immune system and other possibly involved pathological components.

## 2. Methods

**Figure 1:**
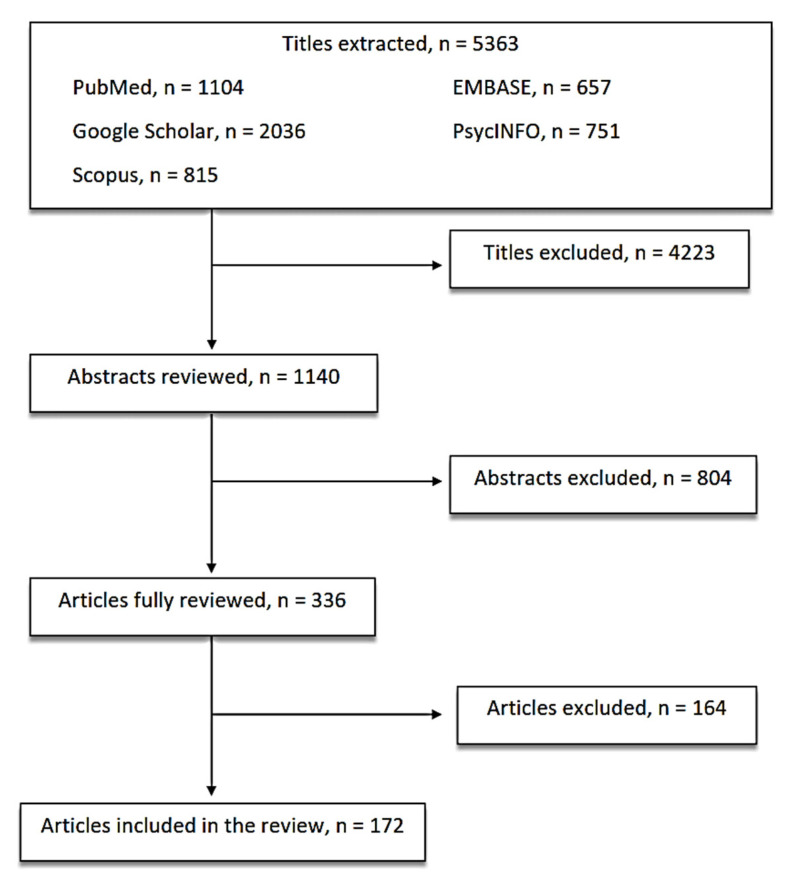
Article Selection Flow Chart.

According to the Preferred Reporting Items for Systematic Reviews and Meta-Analyses (PRISMA) guidelines [34], the databases of PubMed, Scopus, Embase, PsycINFO, and Google Scholar were accessed in order to research and collect articles that were published only in the English language. Free text terms and MeSH headings were combined as follows: “(inflammation OR metabolic markers OR cytokines OR immune system) AND (mood disorders OR bipolar disorders OR depression)”. All of the authors agreed to include in the review conference abstracts, posters, and case reports if published in indexed journals. The following inclusion criteria were adopted: preclinical accurate studies carried out with standard/proven techniques and clinical studies carried out in children/adults with reliable diagnosis of mood disorders, according to structured interviews and standardized criteria and reliable assessment of outcome measures. All of the authors equally contributed to identifying potential information specific to this topic among the titles and abstracts of the publications. The first selection excluded 4223 titles because: a) they were duplicates; b) they did not concern the scope of the paper; c) they were not informative enough. The second selection excluded 804 abstracts after being read and reviewed, as the information reported did not fulfill the scope of our paper and/or the presented information did not seem relevant to the discussed topic. Subsequently, 144 more publications were excluded after being completely read and evaluated, as they did not provide enough information and/or result sufficiently in line with our review. Finally, 172 papers were included in the present review (Figure 1). The search was last updated in December 2019.

## 3. Inflammation, Immune System, and Mood Disorders

Inflammation is a non-specific process, triggered by internal and external stressors that represent a defense response of the body, but may also contribute to the onset of different diseases. This definition, widely accepted for most somatic diseases, also finds applicability in neuropsychiatric disorders, particularly in mood disorders [30,32]. 

The role of inflammation in mood disorders has been widely studied, although it is yet unclear. Several authors have proposed that stressful stimuli can contribute to the development of mood disorders through the activation of inflammatory processes, which may interfere with the physiological 5-HT functioning, the neurotrophins, and the HPA axis [30,32,35,36,37,38,39,40,41]. According to this view, the alteration of the latter mechanisms would be a consequence rather than an etiologic factor of mood disorders [42]. In addition, recent studies highlighted the possible role of proteins defined as "inflammasome" as a molecular bridge between psychological stress and adaptive endogenous responses [40]. In 2013, Iwata proposed the “inflammasome hypothesis of depression” [43]. The inflammasome is a cytosolic protein complex, usually generated in response to infection, but also to many other exogenous and endogenous factors [30,44]. Currently, it is known that the inflammasome is directly involved in a pro-inflammatory state, oxidative stress and, perhaps, in the onset of depressive symptoms [30,44,45,46].

**Figure 2:**
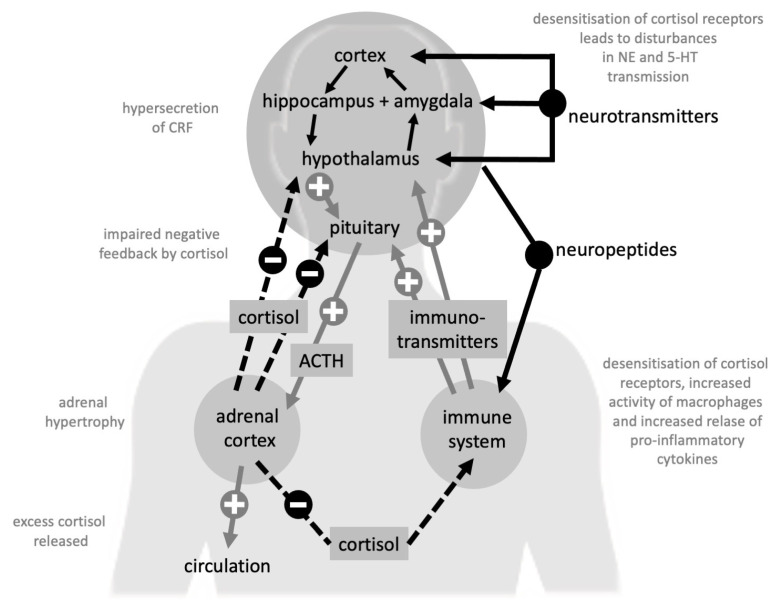
Complex interactions amongst hypothalamus-pituitary-adrenal axis (HPA-axis), neurotransmitters, and immune system. ACTH: adrenocorticotropic hormone; CRF: corticotrophin releasing factor; NE: norepinephrine; 5-HT: serotonin.

Furthermore, in the last two decades, increased blood and cerebrospinal fluid (CSF) pro-inflammatory cytokine concentrations, including interleukin-1β (IL-1β), IL-6, interferon-γ (IFN-γ), and tumor necrosis factor alpha (TNF-α) were revealed in MDD and BDs [35,47,48,49,50,51], leading to the cytokines hypothesis of mood disorders [39] (Figure 2).

It may seem surprising that the activity of the immune system might influence social experience, but some studies suggest that such influence can provide a survival advantage. It is possible that inflammation might increase sensitivity to negative social experiences (in order to safeguard wellbeing in the event of a possible threat) [38,52].

### 3.1. Inflammation in CNS

A mild inflammatory state of the central nervous system (CNS) is considered typical of MDD [53], as demonstrated by post-mortem studies on the brains of depressed patients [54,55]. In particular, several data have associated MDD with microglia and astrocytes activation, together with an increased expression of pro-inflammatory mediators and neurotoxic factors, including superoxide, nitric oxide (NO), and TNF-α [47,48,49,50,51,56,57]. Even in neurodegenerative diseases, neuro-inflammation is associated with an excessive activation of microglia, which provokes harmful effects and may lead to neurotoxic processes [55]. However, the exact causes of the glia activation are still unknown. It was proposed that immune pathway alteration during critical growth phases might prime microglia, while subsequent triggers may spark a severe and prolonged neuro-inflammation, leading to brain damage [55]. Further, it was suggested that systemic inflammation might induce neuro-inflammation through glia activation [58,59,60], since cytokines can pass the blood-brain barrier and stimulate immune and endothelial cells to produce inflammatory mediators [30,32]. Therefore, brain inflammatory activation by external and internal stressful factors could justify the neurodegenerative processes observed in MDD and BDs, as supported by structural alterations observed in several brain areas of depressed and bipolar patients (i.e., prefrontal cortex, hippocampus, and amygdala) [58,59,60]. 

It is known that cytokines long-term exposure can trigger depressive episodes. Many patients undergoing immune-therapy with interferon-α (INF-α) developed depressive clinical pictures, especially considering the sub-threshold syndromes [61,62,63,64]. The administration of pro-inflammatory cytokines, such as IFNα or their inducers (specifically endotoxins or anti-typhoid vaccination) may precipitate depressive symptoms in healthy subjects [65,66,67]. Besides depressive moods, subjects exposed to endotoxin also showed feelings of social disconnection, and increased neural activity related to pain and threats in response to negative social experiences [68,69,70]. On the contrary, in depressed subjects suffering from other diseases, such as rheumatoid arthritis, psoriasis, and cancer, blockade of TNF-type cytokines or other inflammatory components (such as cyclo-oxygenase 2) significantly reduced depressive symptoms [71,72,73,74]. 

### 3.2. Peripheral Inflammation and Depression

Studies in animals support these observations, in that the administration of cytokines or cytokine inducers (i.e., lipopolysaccharide, LPS) caused depressive phenotypes [75,76]. According to some authors, even dysfunctional gut permeability could cause a greater bloodstream translocation of intestinal gram-negative bacteria LPS, with consequent systemic inflammation, as described in cases of prolonged inflammation [77,78,79]. According to this hypothesis, higher serum levels of IgM and IgA against six enterobacteria were revealed in MDD subjects when compared with controls [77,78,79]

As already mentioned, cytokine levels show significant variability in mood disorder patients. The majority of available findings reported elevated levels of pro-inflammatory molecules in subjects affected by mood disorders, including pro-inflammatory cytokines (i.e., IL-4, TNF-α, IL-1β, IL-6), soluble receptors of IL-2 and TNF-α type 1 (sIL-2R and sTNFR1, respectively), and reactive protein C (CRP) [47,48,49,50,51,80], indicating innate immune system dysfunctions. Furthermore, great variability in cytokine profiles was also observed in the different phases of the disease (depression, mania, and euthymia), suggesting variable involvement of inflammatory dysfunctions in different mood states and severity [50]. Currently, the strongest evidence is an association between pro-inflammatory cytokines and depressive episodes [81]. Many inflammatory biomarkers elevated during depressive episodes, but a careful analysis of the literature showed that IL-1β, IL-6, TNF-α, and CRP are the most reliable biological markers. Serum CRP levels were related to decreased motivation and psychomotor retardation [82], as well as to symptoms of anxiety in psychiatric patients [83,84]. Even some polymorphisms of pro-inflammatory cytokine genes, including IL-1β, TNF, and CRP, appeared to be linked with depression and treatment response [85,86,87,88].

Biological markers of inflammation appeared to increase, not only in a subset of depressed patients, but also in people with other neuropsychiatric conditions, such as obsessive–compulsive disorder, anxiety, schizophrenia, and post-traumatic stress disorder (PTSD), putting into question their specificity [39,83,89]. Therefore, it was proposed to verify the impact of inflammation in depression according to specific symptoms described in the research diagnostic criteria (RDCs) of the National Institute of Mental Health. Such symptoms refer to anhedonia, anergy, psychomotor impairment, and increased threat sensitivity with the result of anxiety, excitement, and alarm [83]. The presence of inflammation seems to reduce the response to ADs, as observed in a recent study in which almost half of resistant subjects, with failure response to conventional treatments, showed a CRP level >3 mg/L (indicative of elevated inflammatory status) [90]. Notably, the patient rates with high CRP levels varied according to the examined subjects, being higher in people with a positive history of depression and treatment resistance, child abuse, or other comorbid medical diseases and metabolic syndromes [90]. 

**Figure 3:**
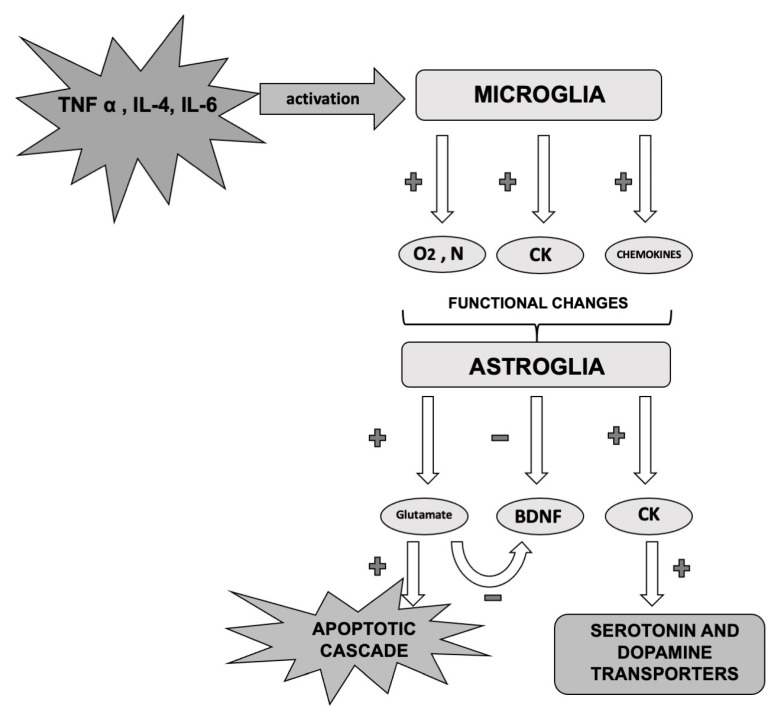
Cascade effects of pro-inflammatory cytokines on microglia and related functional changes. O_2_: oxygen; N nitrogen; CK: cytokines; TNF: tumor necrosis factor; IL: interleukin; BDNF: brain-derived neurotrophic factor.

Finally, T-cells seem to be able to protect laboratory animals from stress and depression [37,91]. By transferring T-cells to chronically stressed animals, an antidepressant phenotype was obtained. This observation was associated with the activity of pro-inflammatory cytokines released by T-cells in the meningeal space, particularly IL-4 [37,91]. Indeed, IL-4 was related to the stimulation of astrocytes BDNF production and the shift of microglia immune responses towards a neuroprotective M2 phenotype, together with an increase in hippocampal neurogenesis [37,91]. Similar results were reported in mice under acute stress conditions, where the migration of T-cells into the choroid plexus (located in the brain ventricles) resulted in an induction of glucocorticoids to the expression of the intercellular adhesion molecule 1 (ICAM1), with reduced anxiety [37,91]. Interestingly, regulatory T (T-reg) cells might also modulate inflammatory pathways and ensuring neuronal support during stress [30,41] (Figure 3).

## 4. Effects of Inflammation on the 5-HT System

According to the monoamine hypothesis, the development of MDD resulted from a reduced, depleted, or dysfunctional monoamine neurotransmission. Different factors can cause an alteration in monoamine activity, such as decreased plasma L-tryptophan (L-TRP) concentrations. The low plasma L-TRP levels revealed in MDD have been related to an increased production of IL-1β, TNF-α, and INF-γ [13,14,15,35], which would promote the degradation pathways of L-TRP.

Indeed, pro-inflammatory cytokines may stimulate inflammatory signaling factors, including the nuclear factor (NF)-κB, p38 mitogen-activated protein kinase (MAP-K), and signal transducer and activator of transcription (STAT). One of the different consequences provoked by these transcription factors is the activation of L-TRP catabolism through the enzyme indoleamine 2,3 dioxygenase (IDO) pathway (the so-called TRYCATs pathway), with the production of various L-TRP catabolites, including kynurenine (KYN), and the reduction of plasma L-TRP concentration [9,13,14,15,35]. As a result, it was suggested that the inflammation-induced increase of L-TRP degradation would be typical of depression and associated with an increase in TRYCAT catabolite production [9,14], rather than a L-TRP/5-HT depletion, as previously supposed. 

Animal models support the involvement of IDO activation in mood disorders pathophysiology [9,15,35]. In a study on mice population, TRYCATs was able to trigger depressive and anxious behaviors [9,14]. In addition, TRYCATs also showed neurotoxic effects: the KYN can be converted into quinolinic acid (QA), a potent N-methyl-D-aspartate (NMDA) agonist, which stimulates glutamate release resulting in lipid peroxidation [9,14,15,35]. The consequences of glucocorticoids imbalance and resistance contribute, in turn, to the continuous secretion of pro-inflammatory cytokines. 

**Figure 4:**
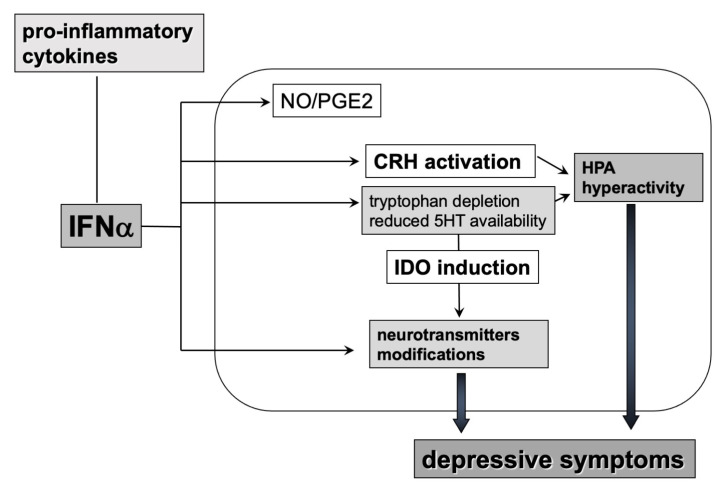
Effects of pro-inflammatory cytokines on 5-HT metabolism, HPA-axis, and oxidative mechanisms. IFN: interferon; CRH: corticotropin-releasing hormone; PGE2: Prostaglandin E_2_; IDO indoleamine 2,3 dioxygenase; NO: nitric oxide.

Again, patients affected by hepatitis C who developed depressive symptoms under IFN-α treatment showed decreased blood L-TRP levels and increased blood KYN concentrations [9,35] (Figure 4).

## 5. Stress-Induced Activation of the Inflammatory Response

Although neuro-inflammation seems to contribute to mood alterations [30,35], the causal factors underlying it are currently unknown. In depressive states associated with inflammatory/immune system diseases, the pathophysiological factors might be the same involved in neuro-inflammation. However, in depression occurring in subjects with no medical comorbidity, stressful life events would play a critical role. Epidemiological studies on clinical and community samples showed a significant association between a history of early and/or recent stressful events and mood disorders [30,35]. Moreover, psychosocial stressors demonstrated to be robust triggers of peripheral and central inflammation, both in animals and in humans [14,15,30,35]. In a study on rats under stressful conditions, increased concentrations of IL-1β and IL-6 were reported in the brain and blood [14,15]. In humans, some studies described increased production of IFNγ and TNFα during stress states [37,38]. Increased levels of CRP were linked to chronic stress conditions, including interpersonal conflicts and perceived stress, [14,15,32,37,38,91], as well as to severe early life events, such as childhood abuse [14,15,30,35]. Further, increased IL-6 and NF-κB response to antigenic challenge and psychosocial stressors were detected in MDD patients [14,15,30,35].

For a long time, HPA dysfunctions have been considered one of the most relevant factors underlying the onset of depression, although their precise mechanisms have not yet been fully elucidated [92,93,94]. In the central nervous system, glucocorticoids participate in the regulation of neuronal survival and neurogenesis, especially at the level of hippocampus, as well as to the modulation of memory processes and emotional learning [95], in addition to regulating, through negative feedback, on limbic and hypothalamic areas, the release of corticotropin-releasing factor (CRF). At the peripheral level, glucocorticoids act by modulating the immune and inflammatory response through specific receptors located on the membranes of lymphocytes and leukocytes [96]. The hypothalamus seems to be the brain area most involved in the inflammation trigger after psychosocial stress [35]. In particular, psychosocial stressors can induce hypothalamic release of catecholamines and CRH that act on the HPA axis. At the level of the immune system, the catecholamines, by increasing NF-κB expression, trigger the production of inflammatory cytokines [30,35] that, in turn, after entering the brain, may cause the activation of microglia and consequent neuro-inflammation [32,35]. On the other hand, the release of CRH activates the HPA axis, followed, in physiological conditions, by the negative feedback on the release of CRH and the inhibition of inflammatory responses. In pathological conditions, as in the case of depression or chronic stress, the presence of a state of “glucocorticoid resistance”, possibly related to cytokines effects on glucocorticoid receptors, may lead to HPA axis hyper-activation and neuro-inflammation perpetuation [35]. Such phenomena would seem to provoke detrimental effects on the brain structures, and may underlie the brain damage possibly associated with MDD and BDs, as they cause monoamine metabolism alterations, decreased neurotrophism, and excitotoxicity [9,10,35]. Different scholars reported increased cortisol levels in plasma, saliva, and urine of depressed patients, in some cases associated with an increased volume of pituitary and adrenal glands [97,98]. Mineralocorticoid receptors (MRs) may also play a role in HPA axis dysfunction in depression, such as the desensitization and loss of inhibition in HPA feedback [92]. The consequences of glucocorticoids imbalance and resistance would contribute to the continuous secretion of pro-inflammatory cytokines, also altering the hippocampal microglia functions [99]. The persistence of cytokine secretion, in turn, may lead to the activation of specific metabolic pathways, such as the shunt of TRP (also known as “kynurenine pathway”), with the production of QA stimulating the glutamatergic system and lowering 5-HT production [100,101]. In addition to inflammation and neurotransmitters, dysfunction of the HPA axis could negatively impact the production of neurotrophins and promote the oxidative stress [92,93,94].

In conclusion, HPA axis hyperactivity, hypercortisolemia, induction of inflammatory patterns (such as the increase in circulating cytokines), stimulation of specific leukocyte populations, alterations of cardiovascular risk factors (such as CRP) and of platelet reactivity, could be part of the same mechanisms of depression.

## 6. Mood Disorders and Oxidative Stress

Different clinical and preclinical studies reported altered levels of oxidative stress markers in depression, such as reduced concentrations of non-enzymatic and enzymatic antioxidants, which can be normalized by AD treatment [102,103,104]. Moreover, some antioxidants (zinc, N-acetylcysteine, omega-3 free fatty acids) show some antidepressant properties [105,106]. The precise mechanisms underlying the altered redox responses in different forms of depression are not completely known. It has been hypothesized that monoaminergic, neurotrophic, and HPA axis dysfunctions may affect metabolic and redox mechanisms in depression. Moreover, a dysfunctional response to stressors may be related to inadequate lifestyle and dietary habits, with consequences on the anti-oxidant response and metabolic or nutritional conditions of depressed patients [107,108,109,110]. The antioxidant system consist of two main components: non-enzymatic antioxidants, such as glutathione, thiols (R-SH), plasma proteins, uric acid (UA), vitamin C, vitamin E, zinc and coenzyme Q10, and enzymatic antioxidants, such as superoxide dismutase (SOD), catalase, glutathione peroxidase and reductase, and thioredoxin system [102,103,104]. The main components protecting against brain damage caused by free radicals are antioxidant enzymes, expressed in both peripheral organs and the brain [102,103,104]. In any case, together with an altered immune-inflammatory response, the induction of oxidative stress, including reactive oxygen species (ROS) and reactive nitrogen species (RNS), appears to play a crucial role in aging and in the development of severe diseases, such as cancer, cardiovascular, and neurodegenerative disorders (e.g., Parkinson’s disease, Alzheimer’s disease and Huntington’s chorea) [111,112,113,114], as well as psychiatric disorders [115,116], including MDD and BDs [117,118,119]. This is possibly due to the high vulnerability of the CNS to oxidative damage [102,103,104]. Indeed, the excessive production of reactive species, that at a low physiological concentration function as signaling molecules, and play a key role in regulating the immune cells activity, can significantly damage different cellular components, including proteins (receptors and enzymes), lipids, and DNA, eventually resulting in apoptosis and cell death. Moreover, ROS and RNS may alter important brain functions throughout the modulation of neurotransmitters, such as the glutamate involved in depression neurobiology [102,110]. In addition, ROS and RNS may act as triggers of autoimmune responses, altering the chemical structure of several molecules with the production of autoimmune epitopes. This is the case of the nitration of proteins that give rise to nitrotyrosine—a highly immunogenic neo-epitope. In the same way, the oxidation of auto-epitopes of fatty acids, normally ignored by immune cells, can lead to their recognition after the damage of the components of the lipid membranes [102,103,104]. In some cases of depression, altered concentrations of non-enzymatic antioxidants were revealed, such as decreased levels of vitamin E and coenzyme Q10. In a group of depressed patients, reduced levels of vitamin C or vitamin A at the baseline were normalized by ADs treatments [104]. However, these data are controversial, since other studies reported opposite findings (increased level of vitamin E and negative relation between vitamin C and severity of depression) [104]. Other studies showed different activity of antioxidants enzymes, including SOD, catalase, and glutathione peroxidase, in patients with MDD compared with healthy subjects. Most of the available data reported an increase of the SOD activity in depression [120], but opposite results were also published [121]. However, ADs might reduce the action of SOD that seems positively related to depression severity. In MDD, also, an increased catalase and glutathione-reductase activity and a decreased of glutathione-peroxidase response were revealed, with a normalization following ADs treatment [120]. However, cases of reduced catalase activity were also described in depression [122]. Again, in a group of subjects with a single depressive episode, the total antioxidant capacity was lower than the oxidative stress index, when compared with healthy subjects, but the values were normalized after the administration of specific treatments [104]. A series of studies reported lipid oxidative damage in depression. A first study showed a decrease in polyunsaturated fatty acids (PUFAs) of red blood cell lipid membranes in depressed patients, indicating an increase in long chain degradation by peroxidation. Indeed, the oxidative potential index (OPI), calculated to measure the tendency of fatty acids to oxidize, was reduced in these subjects, suggesting a decreased oxidation capacity by the phospholipids with a consequent increase in long-chain degradation by peroxidation [105,106]. In acute forms of depression, some authors observed an increase in oxidative stress associated with DNA damage in blood, urine, and brain tissue, highlighted by a higher concentration of the oxidative stress indicator damage 8-hydroxy-guanine (8-OH-Gua) compared with the control group [105,106].

Overall, these data could reflect an altered reactivity of the antioxidant system in different mood disorders that, albeit interesting, require to be substantiated by more specific data [108,123].

## 7. Mood Disorders and Uric Acid

Together with the purines, UA, the final compound of purines catabolism, seems to play a leading role in many cell functions, regulating sleep/wake cycle, appetite, cognitive ability, memory, seizure threshold, social interaction, and impulsiveness [124,125]. Furthermore, purines and UA contribute to modulate energy metabolism and signal transduction, platelets, muscles, and neurotransmission physiology, cell growth, proliferation, and survival [126]. Currently, it is also well known the role of ATP and adenosine (part of its catabolism) in different brain areas, including the cerebral cortex, hypothalamus, and the limbic system [127], where they act as co-modulators of other neurotransmitters, such as γ-aminobutyric acid (GABA), dopamine, glutamate, and 5-HT, all involved in the pathophysiology of mood disorders [127,128,129]. Different enzyme subtypes degrade ATP into adenosine and inosine. Adenosine can be absorbed by the terminal transporters of the nerve and broken down into inosine, transformed, in turn, in hypoxanthine and, finally, cleaved by xanthine oxidase (XO) to generate UA, its downstream product [130]. In physiological conditions, there is a balance between the synthesis and degradation of purines [126]. Over-production of UA proved to play an emerging role in different human diseases [126], including neuropsychiatric disorders [124,125]. In previous years, several studies indicated the possible involvement of purinergic system dysregulations in mood disorders, as well as in neurodegenerative and neuro-inflammatory disorders (i.e., multiple sclerosis, seizures, migraine) often comorbid with depression [124,125,131,132,133,134,135,136,137,138,139]. The correlation between bipolar spectrum disorders with gout and Lesch–Nyhan disease, as well as obesity, thyroid alterations, cardiovascular disease, and diabetes, has been known all along, suggesting an alteration of energy and metabolic processes in mood disorders [124,125,140].

Kraepelin was the first to hypothesize the possible role of the purinergic system in mood disorders [141], and that the excretion of UA in subjects with altered mood states was significantly reduced. Indeed, the purinergic system has been related to MDD, BD, and anxiety symptoms, including dysfunctional sleep, anhedonia, changes in appetite, energy and motor functions levels, as well as cognitive deficits and psychomotor agitation [124]. During the 1970s, Brooks and colleagues [142], basing their hypothesis on the rationale that UA clearance resulting from a significant turnover of purines in the brain, investigated peripheral UA in the attempt to identify a connection to specific mood symptoms. A significant correlation between UA and hallucinations, suicidality, and manic symptoms was observed, and the results led to a subsequent speculation that the purinergic system might be connected to mood dysregulation because of a dysfunction of ATP and/or adenosine due to a hereditary or neuro-inflammatory substrate [142]. More recently, some studies investigated the peripheral levels of potential biomarkers of the purinergic system, particularly UA, in subjects affected by mood disorders. Interestingly, BD and MDD patients showed altered levels of serum UA, with higher concentrations during the manic phase [143,144,145,146] and lower during depressive phases. Furthermore, UA levels in depressed patients seem to normalize after a treatment with ADs [147], some mood stabilizers (lithium [148,149], and carbamazepine [150]). In addition, genetic studies suggest that some purinergic receptors subtypes could be involved in many aspects of behavior and mood dysregulation [148]. Some single-nucleotide polymorphisms showed a significant association with an increased risk of mood disorders [151]. Furthermore, a dysfunction of the downstream adenosine receptor was demonstrated in BD, highlighting an alteration of the levels of cAMP, protein kinase C, and intracellular Ca^2+^ in manic patients. In agreement with this finding, a recent study showed that purinergic modulators seem to rapidly improve the clinical picture of mood disorders [128,145]. In vitro studies showed that ATP and adenosine could be potential targets for future treatments of mood disorders, and two inhibitors of the XO enzyme, allopurinol and febuxostat, showed an antidepressant effect, similar to fluoxetine, a selective serotonin reuptake inhibitor (SSRI) AD, in animal models [151]. 

The evidence that a dysfunctional purinergic system influence a multiplicity of central and peripheral processes supports the hypothesis that mood disorders could be re-considered as systemic diseases. Furthermore, in the view of a unified pathogenetic model of mood disorders, currently it is known that the purinergic system also takes part in neuro-inflammation and neurotoxicity mechanisms [152,153,154,155,156]. Although the processes through which this occurs are still unclear, several studies show that a possible mediator could be represented by oxidative stress [157,158,159,160].

These preliminary data suggest the need to study biochemical mechanisms underlying purinergic dysfunctions, since these system components appear to be promising new therapeutic targets in mood disorders [144,161].

## 8. BDNF and Inflammatory Responses in Mood Disorders

For years, evidence has shown neurotrophin alterations in mood disorders, particularly in depression [162,163,164,165,166]. Most studies on depression reported reduced serum and plasma BDNF levels in depressed patients, compared with controls [167,168,169,170,171,172,173,174,175]. However, elevated plasma neurotrophin levels were also found [176]. An increase of circulating BDNF was associated with depression familiarity [177] mixed state episodes [178], and to ADs administration [179,180,181]. Again, a high baseline BDNF level was associated to a positive response to ADs [182]. For these reasons, peripheral BDNF was proposed as a potential diagnostic, prognostic, and therapeutic biomarker for mood disorders, particularly associated with disease severity and response to AD treatments [181,182,183]. However, results are still inconclusive [184,185,186], as high blood BDNF levels were revealed also in resistant depression subjects under pharmacological treatment [44]. In addition, many factors contributed to limiting peripheral BDNF as a reliable biomarker for mood disorders. Indeed, the BDNF production is ubiquitous in peripheral tissues, this neurotrophin is involved in a variety of body functions [187,188,189,190], and its expression and secretion are influenced by many internal and external factors. Moreover, the connection between peripheral and central BDNF is still currently unknown, and reduced blood BDNF levels have been reported in a multitude of neuropsychiatric and neurodegenerative diseases, as well as in medical diseases, making circulating BDNF a non-specific biomarker [191,192,193,194,195,196,197,198,199,200].

In recent years, research has focused on the possible relationships between neurotrophins, inflammation, and stress axis in the pathogenesis of mood disorders. Recent findings reported that BDNF is a key regulator in the neuro-immune axis regulation [201]. However, its potential mechanism in mood disorders remains unclear [201]. Several available data suggested that inflammatory state, promoted and presumably induced by an altered activation of the HPA axis, might contribute to the development and/or progression of the depressive pathology through an alteration of the neuroplasticity caused by reduced BDNF activity [202,203]. It is well known that inflammatory cytokines affect neuronal development and apoptosis [204,205] and, together with stress, could produce a negative effect on neurogenesis and neuroplasticity [206,207]. In laboratory animals, the infusion of LPS in the substantia nigra induced an anxious and depressed phenotype, together with a decrease of BDNF expression in the hippocampus [208,209]. In rat models, the injection of pro-inflammatory substances led to increased IL-1β, IL-6, and TNF-α expression and decreased BDNF-mRNA expression, particularly in the hippocampus [210,211]. IL-1β is a pro-inflammatory cytokines that seems to influence hippocampal cytogenesis and neurogenesis in two distinct ways: directly, by interacting with its IL-1R1 receptor and activating NF-kB, and indirectly, by stimulating the glucocorticoid secretion in response to environmental stress [202]. 

In humans, patients under treatment with IFN-α showed reduced systemic BDNF levels in combination with increased levels of the cytokines IL-1 and IL-2 [212,213]. Some studies suggested a role of IL-1β in reducing BDNF in patients with depression [212], indicating that an increase in IL-1β concentrations was associated with a decrease in BDNF concentrations [212]. However, it was also proposed that IL-1β could influence not only BDNF concentration, but also BDNF signaling pathways [214]. Therefore, high IL-1β levels could alter the signal deriving from the binding of BDNF with its receptors, causing a sort of BDNF resistance despite its levels being normal or high [215,216]. This could explain the opposite results on the relation between inflammation and neurotrophins, and why in some cases of depression, despite drug treatment, there are high levels of BDNF. According to a recent study, even lipoproteins could decrease brain BDNF levels in the prefrontal regions and in the hippocampus, while increasing them in the nucleus accumbens [206]. 

Finally, as outlined above, an increase in microglia affecting different brain regions was observed in depressed patients. It was also described that microglia could regulate the release of BDNF and reduce the expression of BDNF and its affinity with tyrosine receptor kinase B (TrkB) receptor [201]. Conversely, BDNF can promote glia growth and proliferation [217,218] and contribute to the chronic inflammatory state of the brain and neurotoxicity observed in several mood disorders [69,70,71].

However, despite several ongoing studies, there are still no reliable data on the correlation between inflammatory events and the expression of specific BDNF genes [206].

## 9. Conclusions

Nowadays, there is a general agreement that mood disorders are multifactorial disorders, whose physiopathology include a combination of intertwined genetic and/or acquired factors to define a variety of biological endophenotypes and clinical pictures [30,35]. These factors involve not only the brain, but also the whole organism. As a result, mood disorders are considered as systemic diseases [14,15,35,39]. Therefore, the changes in the monoamine system, described at the dawn of biological hypotheses of depression, are now being progressively integrated within a more complex and comprehensive model of mood disorders, including immune/inflammatory processes, oxidative and nitrosative stress, and changes in peripheral organs and tissues (i.e., the gut) [39,77].

In recent years, the involvement of the immune/inflammatory system in mood disorder development has become increasingly relevant, since many studies demonstrated mutual association between inflammatory diseases/biomarkers and mood alterations. Furthermore, more efforts are now directed towards the interactions between inflammation mechanisms and other systems and processes that, all together, might contribute to the pathophysiology of mood disorders and to their complex clinical features. In particular, inflammatory alterations associated with neurotransmitters and neurotrophins dysfunctions, chronic HPA axis activation, purinergic system abnormalities, and increased oxidative stress. It has also been noted that these factors may have different weights in individual cases, probably influencing the different clinical and prognostic characteristics of the disease, as well as the different response to drug treatment [178,219].

Along this view, biomarkers of every factor, possibly involved in the pathophysiology of mood disorders, could be useful to stratify patients based on the mechanism underlying their clinical picture, and to understand which specific groups could benefit from promising treatments based on new drug targets, such as anti-inflammatory treatments [220,221,222]. 

Therefore, to reach a complete management of mood disorders, it would be necessary to verify all the neurochemical, neurobiological, and metabolic factors that "shape" their presentations, in order to identify potential diagnostic, prognostic, and response biomarkers for treatment. Blood biomarkers seem the most promising [223], being reliable, non-invasive, simple to perform, often inexpensive, and employed to categorize, exactly, the population in line with the disease [224,225]. Such strategies would perhaps represent an important step towards precision medicine in psychiatry.
